# Dynamic Change of Albumin-Bilirubin Score Is Good Predictive Parameter for Prognosis in Chronic Hepatitis C-hepatocellular Carcinoma Patients Receiving Transarterial Chemoembolization

**DOI:** 10.3390/diagnostics12030665

**Published:** 2022-03-09

**Authors:** Po-Ting Lin, Wei Teng, Wen-Juei Jeng, Wei-Ting Chen, Yi-Chung Hsieh, Chien-Hao Huang, Kar-Wai Lui, Chen-Fu Hung, Ching-Ting Wang, Pei-Mei Chai, Chen-Chun Lin, Chun-Yen Lin, Shi-Ming Lin, I-Shyan Sheen

**Affiliations:** 1 Department of Gastroenterology and Hepatology, Linkou Branch, Chang Gung Memorial Hospital, Taoyuan 333, Taiwan; linpoting0101@gmail.com (P.-T.L.); rachel.jeng@gmail.com (W.-J.J.); weiting1972@gmail.com (W.-T.C.); cutebuw@yahoo.com.tw (Y.-C.H.); huangchianhou@gmail.com (C.-H.H.); lincc53@gmail.com (C.-C.L.); lsmpaicyto@gmail.com (S.-M.L.); happy95kevin@gmail.com (I.-S.S.); 2 College of Medicine, Chang Gung University, Taoyuan 333, Taiwan; kwlui@adm.cgmh.org.tw (K.-W.L.); hcf5514@adm.cgmh.org.tw (C.-F.H.); gssunny@cgmh.org.tw (C.-T.W.); p22015@cgmh.org.tw (P.-M.C.); 3 Institute of Clinical Medicine, National Yang-Ming University, Taipei City 11265, Taiwan; 4 School of Traditional Chinese Medicine, College of Medicine, Chang Gung University, Taoyuan 333, Taiwan; 5 Department of Radiology, Linkou Branch, Chang Gung Memorial Hospital, Taoyuan 333, Taiwan; 6 Department of Radiology, Tucheng Composite Municipal Hospital, New Taipei City 236, Taiwan; 7 Department of Nursing, Linkou Branch, Chang Gung Memorial Hospital, Taoyuan 333, Taiwan

**Keywords:** hepatitis, hepatocellular carcinoma, liver, albumin, bilirubin, alpha-fetoprotein, survival

## Abstract

Background and Aims: The Albumin-Bilirubin (ALBI) grade is a good index for liver function evaluation and is also associated with the outcomes of hepatocellular carcinoma patients receiving TACE. However, the correlation between the dynamic change to the ALBI score and clinical outcome is seldom discussed. Therefore, this study aimed to investigate the application of ALBI grade and dynamic change of ALBI grade (delta ALBI grade) after first TACE for prognosis prediction in HCC patients with chronic hepatitis C infection. Method: From January 2005 to December 2015, newly diagnosed naive chronic hepatitis C-hepatocellular carcinoma (CHC-HCC) patients who were treated with TACE as the initial treatment at the Chang Gung Memorial Hospital, Linkou Medical Center, were retrospectively recruited. The pre-treatment host factors, tumor status and noninvasive markers were collected. The Cox regression model was used to identify independent predictors of overall survival and tumor recurrence. Results: Among 613 treatment-naive CHC-HCC patients, 430 patients died after repeated TACE during a median follow-up of 26.9 months. Complete remission after repeated TACE occurred in 46.2% patients, and 208 patients (33.9%) had tumor recurrence, with a median recurrence-free interval of 8.5 months. In Cox regression analysis, ALBI grade II/III (aHR: 1.088, *p* = 0.035) and increased delta ALBI grade (aHR: 1.456, *p* = 0.029) were independent predictive factors for tumor recurrence. Furthermore, ALBI grade II/III (aHR: 1.451, *p* = 0.005) and increased delta ALBI grade during treatment (aHR: 1.436, *p* = 0.006) were predictive factors for mortality, while achieving complete response after repeated TACE (aHR: 0.373, *p* < 0.001) and anti-viral therapy (aHR: 0.580, *p* = 0.002) were protective factors for mortality. Conclusion: Both ALBI and delta ALBI grade are independent parameters to predict survival and tumor recurrence of CHC-HCC patients receiving TACE treatment.

## 1. Introduction

Hepatocellular carcinoma (HCC) is the sixth most common malignant cancer and the fourth most common cause of cancer-related deaths in the world [1]. Chronic viral hepatitis infection (e.g., HBV, HCV) accounts for around 80% of HCC cases [2]. In comparison with other solid cancers, management and prognosis of HCC highly depend on tumor extent and underlying liver functional reserve. The Child-Turcotte-Pugh (CTP) classification system was widely used for decades to assess patient hepatic function. However, the CTP classification is limited by subjectivity in assessing hepatic encephalopathy and ascites [3]. The albumin-bilirubin (ALBI) grade, solely based on albumin and bilirubin, has been proposed for patients with HCC [4]. It can objectively stratify patients with HCC into three risk categories and predict significantly different overall survival (OS) in separate grades. A large collaborative global study further validated the prognostic value of the ALBI grade across all BCLC stages [5].

There have been several studies discussing the prediction of outcome by using images and radiomics for HCC patients receiving TACE [6,7]. However, limited sample size and lack of consensus for the cut-off values in different parameters possibly affected the results. On the other hand, laboratory data, such as for albumin and bilirubin, allow consensus and are easy to record without technical barriers. Thus, this might allow for more convenient and wide use in clinical practice.

For patients not suitable for curative treatment, transarterial chemoembolization (TACE) may provide better loco-regional tumor control and increase patient survival [8,9]. Despite discrepant results of different transarterial treatment modalities [10], TACE remains the standard of care for patients with intermediate stage HCC and therefore is more frequently used in Taiwan [11]. Recent studies have shown that ALBI grade could be a significant prognostic factor in HCC patients undergoing TACE [12,13,14], but the association of ALBI grade and tumor recurrence post-TACE has yet to be fully evaluated. The aim of this study is to investigate the application of not only ALBI grade but also the dynamic change of ALBI grade (delta ALBI grade) after the first TACE treatment for prognosis prediction, including overall survival and tumor recurrence in HCC patients with chronic hepatitis C virus infection (CHC-HCC).

## 2. Patients and Methods

### 2.1. Patient Selection

**Figure 1:**
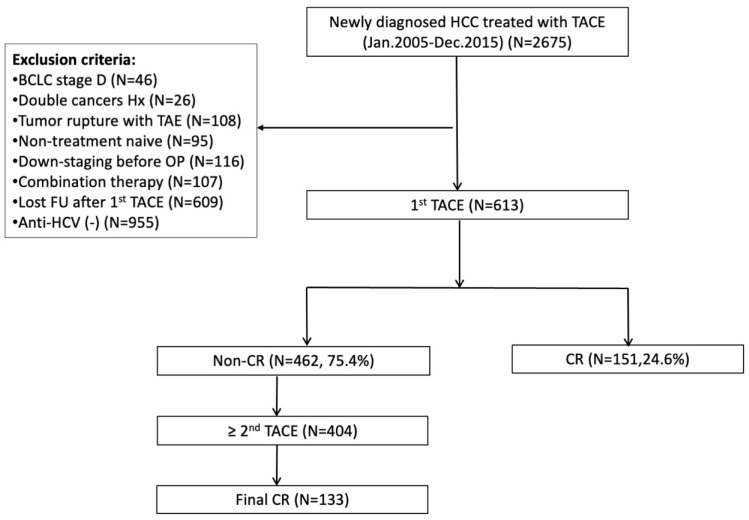
Flowchart of patient recruitment. BCLC, Barcelona clinic liver cancer; CR, complete response; FU, follow up; HCC, hepatocellular carcinoma; HCV, hepatitis C virus; Hx, history; OP, operation; TACE, transarterial chemoembolization; TAE, transarterial embolization.

In Chang Gung Memorial Hospital, a 3000-bed capacity tertiary medical center, chronic hepatitis C–infected patients newly diagnosed with hepatocellular carcinoma and treated with TACE as the initial treatment between January 2005 and December 2015 were recruited (N = 613). The flow chart of the patient selection criteria is shown in Figure 1. The diagnosis of HCC and TACE eligibility were assessed before TACE by contrast-enhanced computed tomography (CT) or magnetic resonance imaging (MRI), which fulfilled the diagnostic criteria according to EASL guidelines [15]. Pretreatment biochemistry data, radiological findings after the first course of TACE treatment, and subsequent follow-up, as well as post-TACE side effects were collected.

### 2.2. Treatment Procedure

All HCC patients were treated according to the BCLC stage [16]. For the TACE procedure, a mixture of 5 mL iodized oil contrast medium, lipiodol, and 10–20 mg adriamycin was infused super-selectively at the level of a subsegmental branch (if possible) or a segmental branch of the feeding arteries, and then embolization was performed using a few gelatin sponge particles [17]. Adiriamycin is used as the protocol in our hospital [18,19]. Sequential TACE was scheduled at 4- to 12-week intervals when a residual viable tumor was detected in the liver at follow-up assessment without appearance of extrahepatic metastases, major portal vein invasion, or deterioration in clinical status. All treatment procedures were performed by an experienced interventional radiologist for dose optimization [20].

### 2.3. Assessment of Treatment Responses Using mRECIST Guidelines

Treatment response was evaluated using the modified response evaluation criteria in solid tumors (mRECIST) [21]. The images obtained before 2010 were re-interpreted by a radiologist, based on the mRECIST. A viable tumor was defined according to the uptake of contrast material in the arterial phase of the dynamic CT or MRI; tumors retaining iodized oil, as well as necrotic lesions without intratumoral arterial enhancement, were regarded as necrotized tumor foci. The objective good response rate referred to the sum of CR and PR. To minimize the possibility of false categorizations, the images were analyzed by two independent experienced radiologists. When a response categorization was not obvious, the final classification was made by the consensus of two radiologists to minimize the variability [22].

### 2.4. Laboratory Methods

Biochemical tests were performed using automated techniques at the clinical pathology laboratories of the hospital. The ULN of serum alanine aminotransferase (ALT) was set at 36 U/L for both males and females. Commercial kits were used for serum anti-HCV detection (Abbott Laboratories, North Chicago, IL, USA) and alpha-fetoprotein (AFP) level (Abbott Laboratories, lower limit of detection: 2 ng/mL). The HCV-RNA levels in this study were measured using a commercial quantitative polymerase chain reaction (PCR) assay, COBAS TaqMan HCV Test (TaqMan HCV; Roche Molecular Systems Inc., Branchburg, NJ, USA, lower limit of detection: 15 IU/mL). The HCV genotype was determined using a genotype-specific probe-based assay in the 5′untranslated region (LiPA; Innogenetics, Ghent, Belgium).

The Albumin-Bilirubin (ALBI) score was calculated using the following formula: linear predictor = (log_10_ bilirubin × 0.66) + (albumin × 0.085), where bilirubin is in umol/L and albumin in g/L; the cut-off points of the ALBI grade were as follows: ≤−2.60 (ALBI grade 1), more than −2.60 to ≤−1.39 (ALBI grade 2), and more than −1.39 (ALBI grade 3) [4]. The ALBI grade was calculated at baseline and after first-cycle TACE or before second-cycle TACE if no CR after first TACE. The delta ALBI grade was calculated by the gradient between post-treatment ALBI grade and baseline ALBI grade. The up-to-7 criteria combined the number of tumors and size of the largest tumor, with the sum being no more than 7 [23].

### 2.5. Follow-Up Protocol

Patients with main portal vein tumor thrombosis, refractory massive ascites, or extrahepatic metastasis were excluded. Overall survival was calculated from the time of the initial TACE treatment to the date of death or last follow-up, and time to recurrence (TTR) was defined as the time from HCC treatment with curative intent to the first disease recurrence documented by radiological assessment. After tentatively curative treatment for HCC with a documented complete radiological response, all patients were followed in specific outpatient clinics. The follow-up protocol included clinical assessment by biochemistry as well as multiphasic CT or MRI every 3 months. HCC recurrence was diagnosed on the basis of combined abnormal findings on AFP level and on one additional dynamic imaging technique confirming hyper-vascularization in the arterial phase with washout in the portal venous or late venous phase. This study was carried out with approval by the Linkou Chang-Gung Memorial Hospital Institutional Review Board (201800831B0), and the IRB waived the signed informed consent due to the retrospective study.

### 2.6. Statistical Analysis

Descriptive data with normal distribution were reported as mean ± standard deviation (SD) or as percentage otherwise as median (interquartile Range (IQR)). The independent Student’s t test and Mann–Whitney U test were used to assess differences between groups in normal distributed and non-normal distributed groups separately. Chi-square was used for categorical variables between the 2 groups. The Kaplan–Meier method was used to estimate survival and recurrence rates. The log-rank test was used to compare survival and recurrence curves between patient groups. A stepwise Cox regression model was used to determine the correlation of the predictive factors and clinical outcomes. A 2-tailed *p* value < 0.05 was considered as statistically significant. Statistical analysis was done using SAS version 9.4.

## 3. Results

### 3.1. Baseline Characteristics and Follow-Up Results

Of these 613 CHC infected HCC patients, the median age was 67.2 (IQR 60.4–74.1) years, and 368 (60.0%) were males. The majority of patients were ALBI grade II (N = 425, 69.3%) and BCLC stage B/C (N = 363, 59.2%) at baseline. Eighty-three (53.9%) patients (including 41 CR and 42 PR) in ALBI grade I, while 230 (54.1%) patients (including 110 CR and 120 PR) in ALBI grade II/III achieved objective response after the first TACE treatment. Two hundred and ninety-eight (48.6%) patients had an increased ALBI grade after the first TACE treatment. Overall, two hundred and eighty-four (46.3%) patients achieved final complete response after repeated TACE, and more than half of them (71.6%) received two or fewer TACE treatments. Among the patients achieving final complete response, 208 patients (73.2%) had tumor recurrence during follow-up, with a median recurrence-free interval (RFI) of 8.5 (IQR 4.4–14.0) months. The 1 and 2 year cumulative incidences of tumor recurrence were 46.5% and 64.8%, respectively. Four hundred and thirty patients (70.1%) died during follow-up, with a median overall survival (OS) time of 26.9 (IQR 16.1–43.6) months. Most patients died of hepatic failure (N = 241, 56.0%), and others died of sepsis (N = 103, 24.0%) and gastrointestinal tract bleeding (N = 86, 20.0%).

### 3.2. Determining the Risk Factors Affecting Mortality

Compared with the survival group, patients with mortality had worse tumor stage status (BCLC stage B/C: 63.2% vs. 49.7%, *p* = 0.0009), advanced baseline ALBI grade status (ALBI grade II/III: 77.4% vs. 68.8%, *p* = 0.0450), higher proportion of increased delta ALBI grade after the first TACE (53.3% vs. 37.7%, *p* = 0.0001), less anti-viral therapy use (11.2% vs. 39.3%, *p* < 0.0001), less proportion of within up-to-seven (61.6% vs. 74.9%, *p* = 0.0016), greater bilobar tumor extent (46.7% vs. 31.7%, *p* = 0.0007), and lower complete response rate (35.8% vs. 71.0%, *p* < 0.0001) (Table 1).

The results of multivariate Cox regression analysis showed that pre-TACE ALBI grade II/III (adjusted HR: 1.451 (95% CI: 1.119–1.882), *p* = 0.005), and increased delta ALBI grade after the first TACE (adjusted HR: 1.436 (95% CI: 1.107–1.864), *p* = 0.006) were independent factors related to mortality, while anti-viral therapy (adjusted HR: 0.580 (95% CI: 0.410–0.819), *p* = 0.002) and achieving complete response (adjusted HR: 0.373 (95% CI: 0.123–0.471), *p* < 0.001) were protective factors from mortality (Table 2).

**Figure 2:**
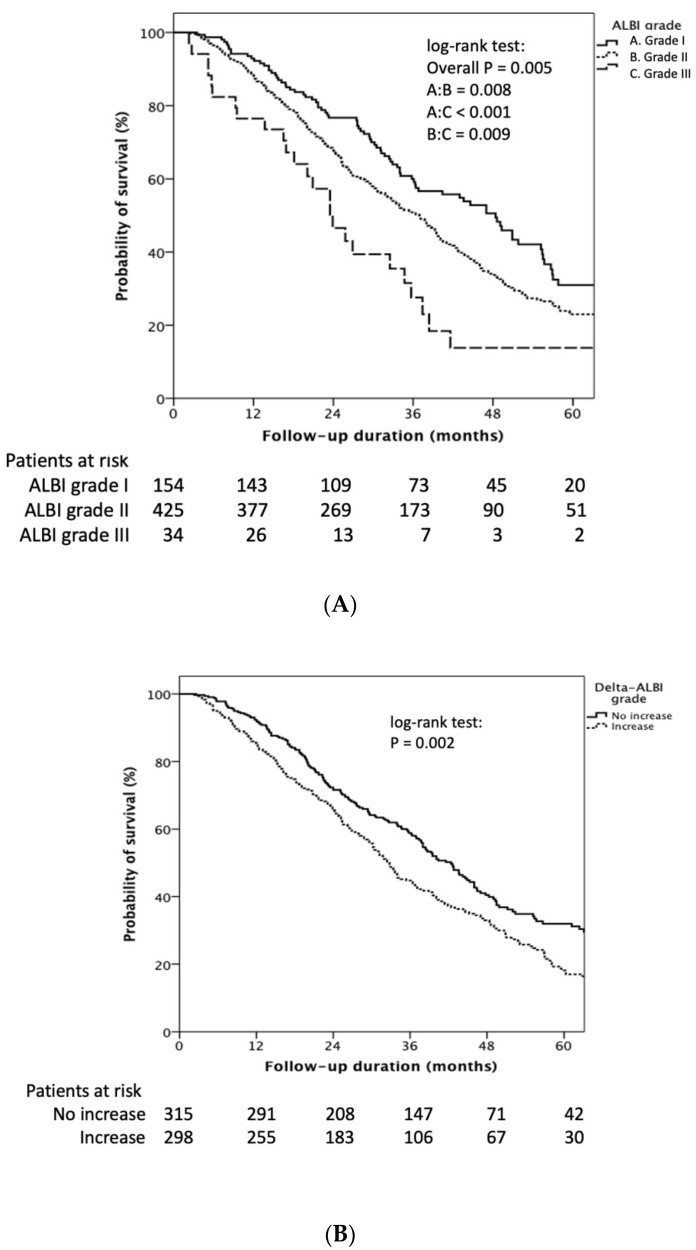
Kaplan–Meier estimates of survival rate stratified by ALBI grade and delta ALBI grade. (**A**) Patients with baseline ALBI grade I had longer OS than grade II/III (median 48.4 (95% CI: 40.9–55.9) vs. 36.9 (95% CI: 33.4–40.4) vs. 23.9 (95% CI: 17.9–29.9) months, respectively, log rank *p* = 0.005). (**B**) Patients who encountered an ALBI grade increase after the first TACE treatment showed significantly decreased OS compared to those who had the same or a decreased ALBI grade (32.6 (95% CI: 29.6–35.6) vs. 42.4 (95% CI: 38.6–46.2) months, log rank *p* = 0.002).

Patients with baseline ALBI grade I had longer OS than grade II/III (median 48.4 (95% CI: 40.9–55.9) vs. 36.9 (95% CI: 33.4–40.4) vs. 23.9 (95% CI: 17.9–29.9) months, respectively, Log rank *p* = 0.005) (Figure 2A). The cumulative 1-, 3-, and 5-year OS rates of ALBI grade I vs. ALBI grade II/III were 93.0%, 60.0%, and 32.0%, vs. 88.0%, 49.0%, and 23%, respectively (*p* = 0.025). Patients who encountered an ALBI grade increase after the first TACE treatment showed significantly decreased OS compared to those who maintained the same or had an decreased ALBI grade (32.6 (95% CI: 29.6–35.6) vs. 42.4 (95% CI: 38.6–46.2) months, log rank *p* = 0.002) (Figure 2B).

The cumulative 1-, 3-, and 5-year OS rates of increased delta ALBI grade vs. not increased delta ALBI grade were 86.0%, 44.0%, and 18.0% vs. 92.0%, 59.0%, and 32%, respectively (*p* = 0.001). Furthermore, in patients with baseline ALBI grade II, delta ALBI grade increase after treatment showed significantly worse OS than those whose ALBI grade was not increased (29.8 (95% CI: 24.7–34.9) vs. 40.1 (95% CI: 36.6–43.5) months, Log rank *p* < 0.001) (Supplementary Figure S1A). On the other hand, in patients with baseline ALBI grade I, delta ALBI grade showed no significant difference in OS between patients with and without delta ALBI grade change (log-rank *p* = 0.529) (Supplementary Figure S1B).

### 3.3. Determining the Risk Factors Affecting Tumor Recurrence

Compared with the non-tumor recurrence group, patients encountering tumor recurrence had advanced fibrotic status (ALBI grade II/III: 76.9% vs. 71.1%, *p* = 0.0453), higher proportion of increased delta ALBI grade after first TACE (51.4% vs. 43.4%, *p* = 0.0413), less anti-viral therapy use (23.1% vs. 35.5%, *p* = 0.0461), and higher mortality rate (61.5% vs. 34.2%, *p* < 0.0001) (Table 3).

The results of the multivariate Cox regression analysis show that baseline ALBI grade II/III (adjusted HR: 1.088 (95% CI: 1.019–1.196), *p* = 0.035) and increased delta ALBI grade after the first TACE (adjusted HR: 1.456 (95% CI: 1.087–2.148), *p* = 0.029) were independent factors related to tumor recurrence (Table 4).

**Figure 3:**
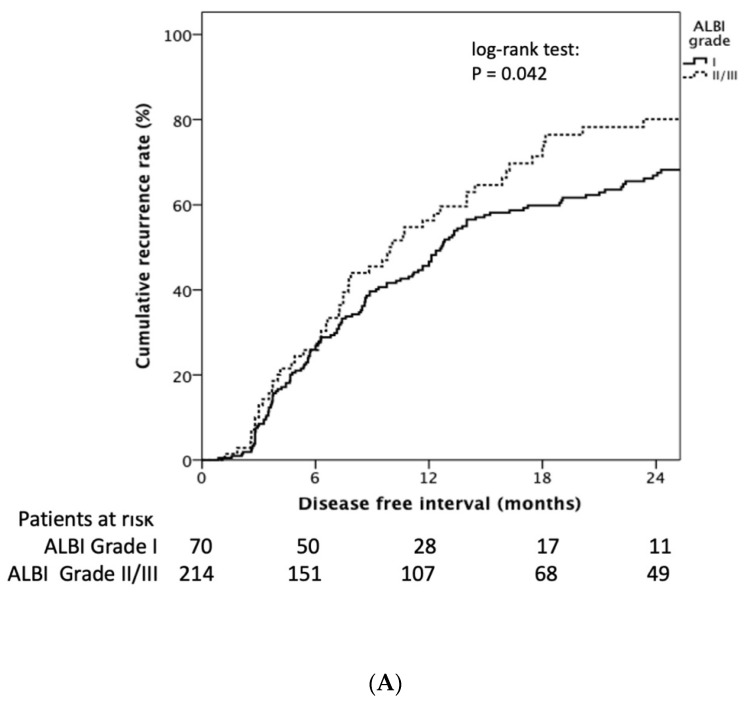

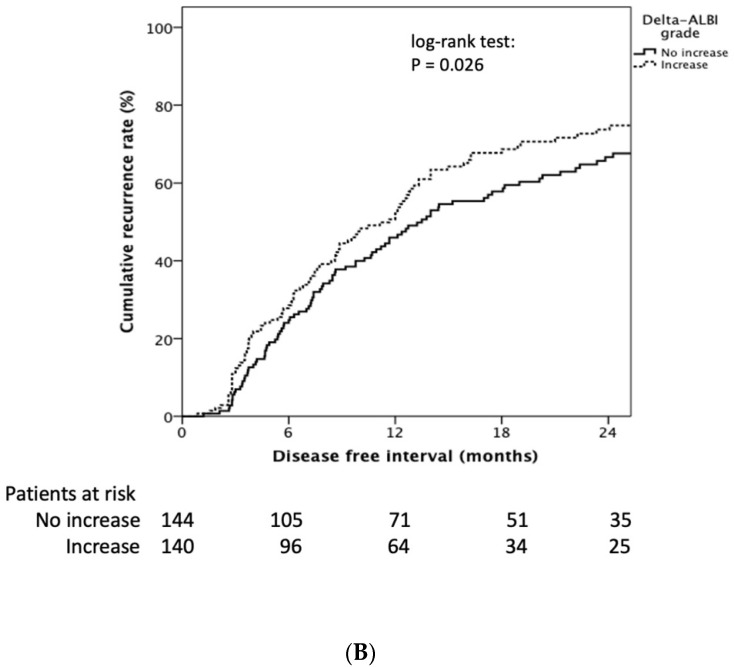
Kaplan–Meier estimates of recurrence rate stratified by ALBI grade and delta ALBI grade. (**A**) Patients with baseline ALBI grade I had longer RFI than grade II and III (median 12.7 (95% CI: 11.2–14.2) vs. 9.9 (95% CI: 6.3–13.6) months, log rank *p* = 0.042). (**B**) Patients encountering an ALBI grade increase after the first TACE treatment showed significantly decreased RFI compared to those who had the same or a decreased ALBI grade (11.7 (95% CI: 8.9–14.4) vs. 13.5 (95% CI: 10.6–16.4) months, log rank *p* = 0.026).

Patients with baseline ALBI grade I had longer RFI than grade II and III (median 12.7 (95% CI: 11.2–14.2) vs. 9.9 (95% CI: 6.3–13.6) months, Log rank *p* = 0.042) (Figure 3A). The cumulative 1- and 2-year tumor recurrence rates of ALBI grade I vs. ALBI grade II/III was 45.0% and 67.0% vs. 56.0% and 79%, respectively (*p* = 0.049). Patients encountering an ALBI grade increase after the first TACE treatment showed significantly decreasing RFI compared to those still had the same or decreased ALBI grade (11.7 (95% CI: 8.9–14.4) vs. 13.5 (95% CI: 10.6–16.4) months, log-rank *p* = 0.026) (Figure 3B).

The cumulative 1- and 2-year tumor recurrence rates of increased delta ALBI grade vs. not increased delta ALBI grade were 50.0% and 73.0% vs. 46.0% and 67%, respectively (*p* = 0.031). Moreover, in patients with baseline ALBI grade I, delta ALBI grade increase after treatment showed significantly shorter RFI than those whose ALBI grade was not increased (7.8 (95% CI: 6.0–9.5) vs. 20.1 (95% CI: 15.0–25.2) months, log-rank *p* = 0.001) (Supplementary Figure S2A). By comparison, in patients with baseline ALBI grade II, delta ALBI grade showed no significant difference of RFI between patients with and without delta ALBI grade change (log-rank *p* = 0.356) (Supplementary Figure S2B).

## 4. Discussion

The current retrospective study aimed to investigate the predictive factors for the clinical outcome of CHC-HCC patients after receiving TACE treatment. In our study, baseline ALBI as well as delta ALBI grade after the first TACE treatment are independent factors to predict tumor recurrence and overall survival for CHC-HCC patients. Furthermore, patients with anti-viral treatment and achieving complete response after repeated TACE treatments had better survival outcomes.

TACE is widely used for patients with hepatocellular carcinoma who are unsuitable for curative treatment. Nevertheless, the characteristics of tumors with a high recurrence rate might interfere with a patient’s treatment choice and survival. Consistent with a previous report [24], despite the fact that some HCC patients can achieve complete response after TACE treatment, recurrence rate remained more than 50%. Hence, a large proportion of patients ultimately face the condition of tumor progression or recurrence. Therefore, this study highlights the importance of exploring new predictive factors for tumor recurrence and the survival benefits for patients with TACE treatment.

TACE is the standard of treatment among intermediate-stage HCC patients; however, the heterogeneity of this group presents a challenge in the choice of best treatment for these patients. Many scoring systems have been studied for predicting the effects of TACE [25,26,27,28,29]; however, the predictive value of these scoring systems has not been well validated, and not all systems are adopted in current practical guidelines. In the current study, ALBI grading was an independent predictive parameter for clinical and survival outcomes among CHC-HCC patients under TACE therapy. ALBI score, which is based on only two factors, serum albumin and bilirubin level, is simpler and more objective than the Child-Pugh classification [30]. It has recently attracted attention by serving as a useful parameter to predict the prognosis and the reserve liver function of HCC patients under different treatment modalities [13,31,32,33]. Furthermore, post-TACE liver decompensation is one of the most serious complications after this procedure and is associated with significant mortality [34]. Though several studies assessing the ALBI score as a predictor for the outcome of patients with HCC have already been published, dynamic change in ALBI has seldom been discussed. Change in the ABLI score has been shown to correlate with the treatment response in HCC patients receiving lenvatinib [35], but it was inadequate to evaluate overall survival due to a short observation period and was also not evaluated for patients receiving TACE treatment. Hiraoka et al. reported that downgrading of the ALBI score indicated poor prognosis for patients with TACE treatment, but the number of patients in this study was limited [36]. The current study represents the largest cohort to date, with an adequate follow-up period to validate the clinical use of dynamic change in ALBI score for CHC-HCC patients receiving TACE treatment. Furthermore, in patients with better reserved liver function, delta ALBI gradient can help to differentiate patients who are prone to encounter tumor recurrence. On the other hand, in patients with worse liver function, the delta ALBI gradient can help to identify patients who are prone to mortality after treatment. Integrating these two parameters into a treatment algorithm might avoid unnecessary treatment and allow prompt switching of therapeutic modality in time to achieve survival benefits. Importantly, patients who experienced the elevation of ALBI score after the first treatment should be reevaluated for the reversible cause, to preserve liver function before the next round of TACE.

Apart from a patient’s ALBI score, patients with anti-viral therapy use and achieving complete response after repeated TACE treatment also achieved better survival outcomes. Interferon-based therapy in combination with ribavirin (RBV) was most widely used in this cohort, owing to the reimbursement policy and the period of time during patient enrollment. Successful eradication of chronic HCV infection with the pegylated interferon-based regimen (Peg-IFN) has proven to improve overall survival by preventing worsening of compensated cirrhosis, the development of HCC (“secondary prevention”) [37], and recurrence of HCC (“tertiary prevention”) [17,38]. In the current study, anti-viral therapy with Peg-IFN appeared to prevent tumor recurrence, but anti-viral therapy did not achieve statistical significance in multivariate analysis because patients without sustained virologic response (SVR) were not excluded. Furthermore, the tertiary prevention effects of direct-acting antiviral agent (DAA) treatment in HCC patients remain debatable [39,40,41,42,43]. Thus, further large prospective studies are needed to clarify the benefit of DAA treatment in HCC patients. On the other hand, tumor-related dynamic changes like tumor response after TACE are important for patient prognosis [44,45]; this has been used for scoring systems such as the ART score and ABCR score [25,26]. In our study, radiological response after TACE treatment also served as a protective predictor for survival. However, different assessment criteria were used for the ART score and ABCR score, which showed dissimilar results in the different cohorts [25,46,47]. Therefore, there is a need to validate our findings in a future study by using the mRECIST criteria, which is a standard tool for measurement.

This study has a few limitations. First of all, it was a retrospective study from a single medical center, and clinical data that might determine treatment response, such as baseline IL-28B polymorphism and the decline of HCV-RNA level at different times, could not be provided. Furthermore, a large number of patients were omitted from the study due to loss during follow-up, which might affect the predictive power in the retrospective setting. Second, only patients with HCV infection were enrolled. Third, an improved diagnostic algorithm for HCC using the advanced diagnostic modality would change the composition of the different stages of HCC [48,49]. Hence, we should be cautious applying ALBI and delta ALBI to other populations. Finally, post-TACE treatment modalities were not addressed, which might have affected the clinical outcome as well. Findings from a prospective study are needed before definite conclusions can be made.

In conclusion, baseline ALBI as well as delta ALBI grade after the first TACE treatment are independent factors for predicting tumor recurrence and overall survival for the CHC-HCC patients. Furthermore, patients with anti-viral treatment and achieving complete response after repeated TACE treatments achieved better survival outcome.

## Figures and Tables

**Table 1:** Baseline clinical characteristics of patients with overall mortality vs. non-mortality.

Variables	All (n = 613)	Mortality		*p*-Value
		No. (N = 183, 29.9%)	Yes (N = 430, 70.1%)	
Age (years)	67.2 (60.4–74.1)	66.7 (61.1–72.7)	67.5 (60.3–74.5)	0.5140
Gender (male, %)	368 (60.0)	99 (54.1)	269 (62.6)	0.0584
BCLC stage 0/A/B/C, n (%)	28/222/240/123 (4.6/36.2/39.2/20.0)	14/78/52/39 (7.7/42.6/28.4/21.3)	14/144/188/84 (3.3/33.5/43.7/19.5)	0.0009
ALBI grade I/II/III, n (%)	154/425/34 (25.1/69.3/5.6)	57/117/9 (31.2/63.9/4.9)	97/308/25 (22.6/71.6/5.8)	0.0450
ALBI grade increase, n (%)	298 (48.6)	69 (37.7)	229 (53.3)	0.0001
Anti-viral therapy, n (%)	120 (19.6)	72 (39.3)	48 (11.2)	<0.0001
NLR	1.72 (1.26–2.55)	1.67 (1.19–2.39)	1.74 (1.27–2.66)	0.2643
Total bilirubin (mg/dL)	0.80 (0.60–1.20)	0.80 (0.50–1.10)	0.90 (0.60–1.30)	0.0067
AST (U/L)	62 (42–98)	62 (37–94)	63 (43–99)	0.1696
ALT (U/L)	56 (33–88)	55 (30–87)	57 (33–89)	0.3461
APRI	1.92 (1.04–3.39)	1.89 (0.86–3.25)	1.92 (1.11–3.39)	0.4968
FIB-4	5.82 (3.78–9.45)	5.25 (3.45–9.84)	5.95 (3.89–9.39)	0.3092
Albumin (g/dL)	3.55 (3.20–3.90)	3.64 (3.30–3.97)	3.50 (3.18–3.88)	0.0129
AFP (ng/mL)	42 (11–297)	40 (9–188)	45 (11–422)	0.1238
Platelet (1000/μL)	99 (69–146)	102 (67–150)	99 (70–143)	0.9431
Tumor numbers > 3, n (%)	185 (30.2)	33 (18.0)	152 (35.4)	<0.0001
Target lesion size (cm)	3.3 (2.1–5.0)	3.0 (2.0–4.5)	3.4 (2.1–5.3)	0.0453
Within up-to-7, n (%)	402 (65.6)	137 (74.9)	265 (61.6)	0.0016
Tumor extent unilobar, n (%)	354 (57.8)	125 (68.3)	229 (53.3)	0.0007
Macrovascular invasion, n (%)	59 (9.6)	15 (8.2)	44 (10.2)	0.5495
Final CR, n (%)	284 (46.3)	130 (71.0)	154 (35.8)	<0.0001
Recurrence, n (%)	208 (73.2)	80 (61.5)	128 (83.1)	<0.0001
Follow-up duration (months)	31.7 (19.2–46.0)	36.9 (27.4–49.3)	26.9 (16.1–43.6)	<0.0001

Abbreviations: AFP, alpha-fetoprotein; ALBI, albumin-bilirubin index; ALT, alanine aminotransferase; APRI, AST-platelet ratio index; AST, aspartate aminotransferase; BCLC, Barcelona Clinic Liver Cancer; CR, complete response; FIB-4, fibrosis-4; NLR, neutrophil-lymphocyte ratio.

**Table 2:** Cox regression of risk factors associated with mortality in HCV patients.

Variables		Crude HR	95%CI	*p*-Value	Adjusted HR	95%CI	*p*-Value
ALBI in baseline	I	Referent			Referent		
	II/III	1.301	1.037–1.631	0.023	1.451	1.119–1.882	0.005
Sex	Female	Referent					
	Male	1.210	0.995–1.472	0.0764			
Delta ALBI grade	No increase	Referent			Referent		
	Increase	1.297	1.013–1.661	0.040	1.436	1.107–1.864	0.006
Anti-viral therapy	No	Referent			Referent		
	Yes	0.502	0.370–0.681	<0.001	0.580	0.410–0.819	0.002
Up-to-seven	Within	Referent			Referent		
	Beyond	1.624	1.338–1.970	<0.001	1.214	0.975–1.511	0.083
Tumor extent	Unilobe	Referent			Referent		
	Bilobe	1.434	1.186–1.734	<0.001	1.107	0.894–1.372	0.351
MVI	No	Referent					
	Yes	1.214	0.715–2.061	0.4726			
CR	No	Referent			Referent		
	Yes	0.347	0.283–0.425	<0.001	0.373	0.123–0.471	<0.001

Abbreviations: ALBI, albumin-bilirubin index; CR, complete response; MVI, macrovascular invasion.

**Table 3:** Baseline clinical characteristics of patients with overall recurrence vs. non-recurrence.

Variables	All (n = 284)	Recurrence		*p*-Value
		No (N = 76, 26.8%)	Yes (N = 208, 73.2%)	
Age (years)	66.2 (60.3–73.0)	66.2 (61.1–75.6)	66.2 (60.0–72.5)	0.2601
Gender (male, %)	156 (54.9)	41 (54.0)	115 (55.3)	0.8931
BCLC stage 0/A/B/C, n (%)	24/144/73/43 (8.5/50.7/25.7/15.1)	6/41/15/14 (7.9/54.0/19.7/18.4)	18/103/58/29 (8.7/49.5/27.9/13.9)	0.4780
ALBI grade I/II/III, n (%)	70/201/13 (24.7/70.8/4.5)	22/50/4 (28.9/65.8/5.3)	48/151/9 (23.1/72.6/4.3)	0.0453
ALBI grade increase, n (%)	140 (49.3)	33 (43.4)	107 (51.4)	0.0413
Anti-viral therapy, n (%)	75 (26.4)	27 (35.5)	48 (23.1)	0.0461
NLR	1.68 (1.24–2.34)	1.85 (1.26–3.08)	1.65 (1.22–2.21)	0.2125
Total bilirubin (mg/dL)	0.80 (0.60–1.20)	0.85 (0.50–1.30)	0.80 (0.60–1.20)	0.4805
AST (U/L)	62 (41–93)	55 (34–88)	62 (46–94)	0.1517
ALT (U/L)	56 (33–90)	40 (24–72)	63 (37–92)	0.0001
APRI	1.95 (1.09–3.50)	1.71 (0.79–3.24)	2.05 (1.32–3.51)	0.1284
FIB-4	5.90 (3.89–9.67)	5.47 (3.89–10.8)	6.36 (3.89–9.42)	0.7924
Albumin (g/dL)	3.60 (3.26–3.89)	3.66 (3.30–3.95)	3.47 (3.20–3.74)	0.0179
AFP (ng/mL)	24 (9–127)	27 (9–205)	23 (9–112)	0.6332
Platelet (1000/μL)	92 (66–137)	96 (66–151)	90 (65–128)	0.2305
Tumor numbers > 3, n (%)	46 (16.2)	8 (10.5)	38 (18.3)	0.1458
Target lesion size (cm)	2.8 (2.0–4.0)	3.1 (2.0–4.3)	2.7 (2.0–3.9)	0.1489
Within up-to-7, n (%)	236 (83.1)	59 (77.6)	177 (85.1)	0.1536
Tumor extent unilobar, n (%)	209 (73.6)	61 (80.3)	148 (71.2)	0.1315
Macrovascular invasion, n (%)	26 (9.2)	10 (13.2)	16 (7.7)	0.1674
Mortality, n (%)	154 (54.2)	26 (34.2)	128 (61.5)	<0.0001
Follow-up duration (months)	37.0 (25.5–52.9)	33.6 (19.1–43.8)	39.6 (29.2–56.6)	0.0003

Abbreviations: AFP, alpha-fetoprotein; ALBI, albumin-bilirubin index; ALT, alanine aminotransferase; APRI, AST-platelet ratio index; AST, aspartate aminotransferase; BCLC, Barcelona Clinic Liver Cancer; CR, complete response; FIB-4, fibrosis-4; NLR, neutrophil-lymphocyte ratio.

**Table 4:** Cox regression of risk factors associated with recurrence in HCV patients.

Variables		Crude HR	95%CI	*p*-Value	Adjusted HR	95%CI	*p*-Value
ALBI in baseline	I	Referent			Referent		
	II/III	1.352	1.015–1.835	0.036	1.088	1.019–1.196	0.035
Sex	Female	Referent					
	Male	1.240	0.978–1.572	0.0756			
Delta ALBI grade	No increase	Referent			Referent		
	Increase	1.558	1.071–2.268	0.020	1.456	1.087–2.148	0.029
MVI	No	Referent					
	Yes	0.898	0.599–1.346	0.6018			
Anti-viral therapy	No	Referent			Referent		
	Yes	0.765	0.517–0.978	0.045	0.983	0.592–1.183	0.321

Abbreviations: ALBI, albumin-bilirubin index; MVI, macrovascular invasion.

## Data Availability

The data that support the findings of this study are available from the corresponding author upon reasonable request.

## Supplementary Materials

The following supporting information can be downloaded at: https://www.mdpi.com/article/10.3390/diagnostics12030665/s1, Figure S1: Kaplan–Meier estimates of survival rate stratified by delta ALBI grade in ALBI grade II (S1A) and ALBI grade I (S1B) patient; Figure S2: Kaplan–Meier estimates of recurrence rate stratified by delta ALBI grade in ALBI grade I (S2A) and ALBI grade II (S2B) patient.

## Abbreviations

AFP, alpha-fetoprotein; ALBI, albumin-bilirubin index; ALT, alanine aminotransferase; APRI, AST-platelet ratio index; AST, aspartate aminotransferase; BCLC, Barcelona Clinic liver cancer; CHC, chronic hepatitis; CR, complete response; CT, computed tomography; CTP, Child-Turcotte-Pugh; FIB-4, Fibrosis-4; HCC, hepatocellular carcinoma; HCC, hepatocellular carcinoma; HBV, hepatitis B virus; HCV, hepatitis C virus; IQR, interquartile range; mRECIST, modified response evaluation criteria in solid tumors; MRI, magnetic resonance imaging; NLR, neutrophil-lymphocyte ratio; OS, overall survival; PD, progression disease; PR, partial response; RFI, recurrence-free interval; SD, stable disease; TACE, transarterial chemoembolization; TTR, time to recurrence.
