# Supplementary material for: Dynamic Change of Albumin-Bilirubin Score Is Good Predictive Parameter for Prognosis in Chronic Hepatitis C-hepatocellular Carcinoma Patients Receiving Transarterial Chemoembolization
Source: Diagnostics (Basel). 2022 Mar 9;12(3):665. doi: 10.3390/diagnostics12030665 (PMC8947376; doi:10.3390/diagnostics12030665)
Supplement: Supplementary file 1 [file diagnostics-12-00665-s001.zip › diagnostics-1590428-supplementary.pdf]

Supplementary Figure S1A.

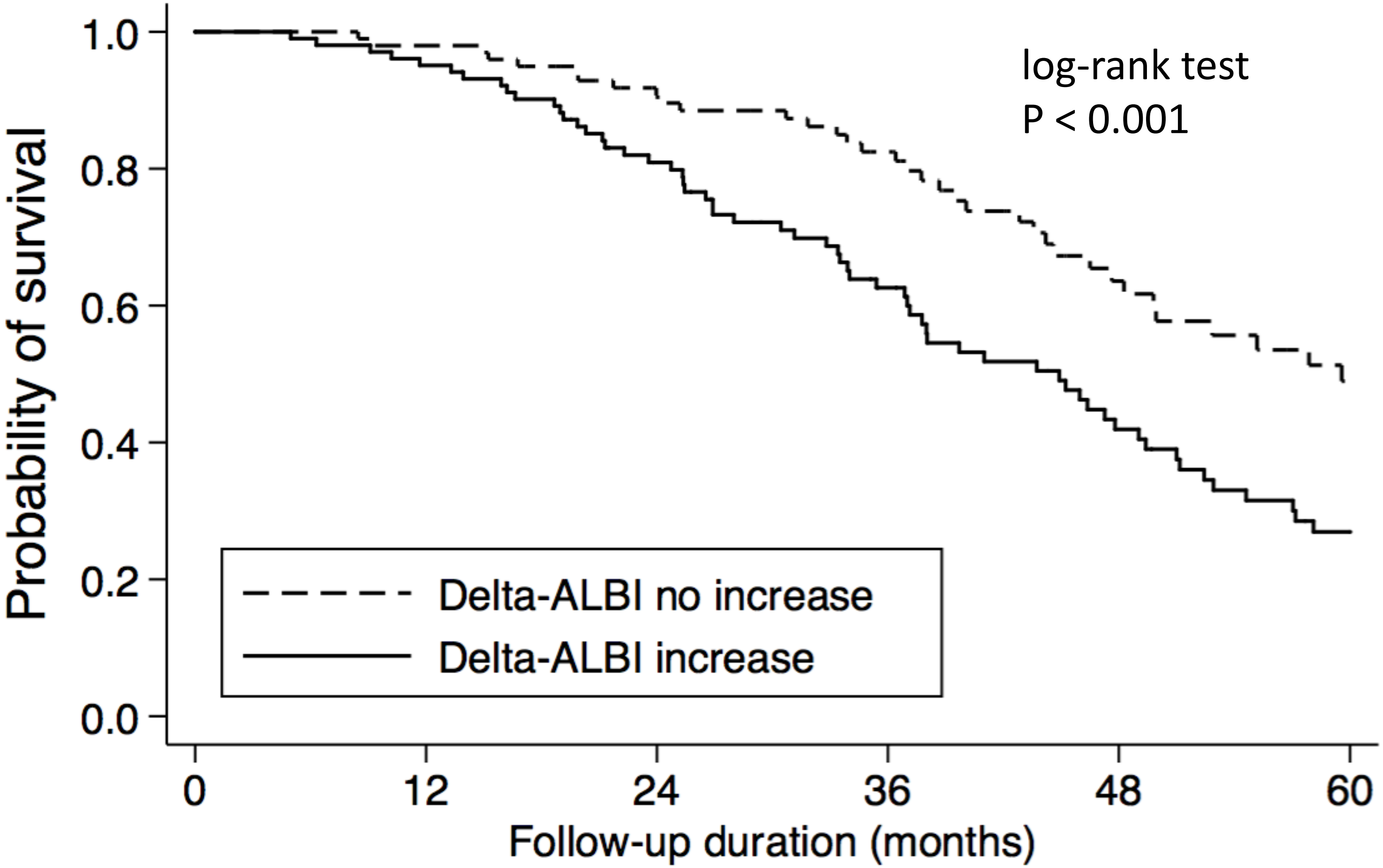

Number at risk						
Delta-ALBI no increase	99	97	82	62	34	21
Delta-ALBI increase	102	97	75	49	29	17

Supplementary Figure S1B.

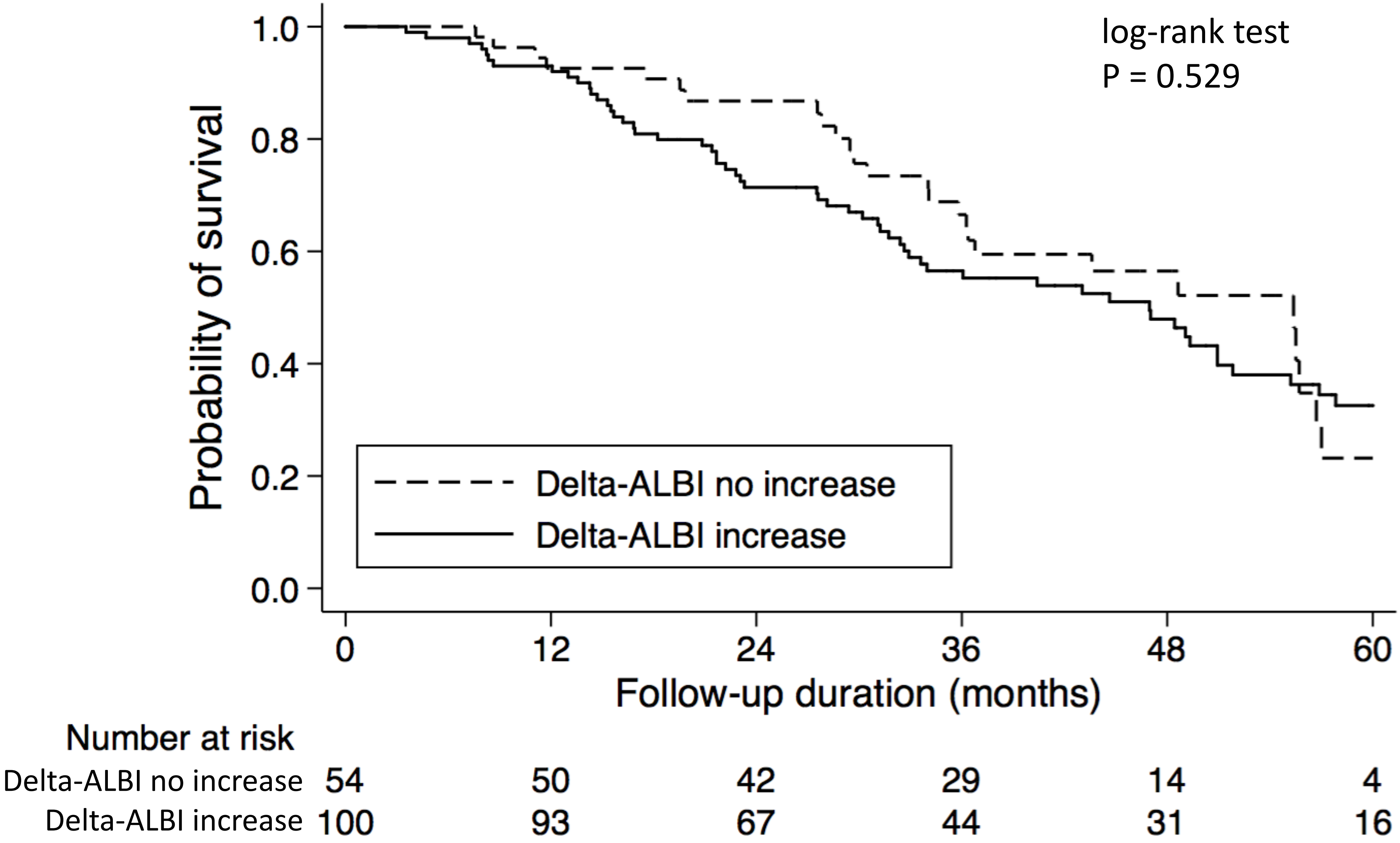

Supplementary Figure S2A.

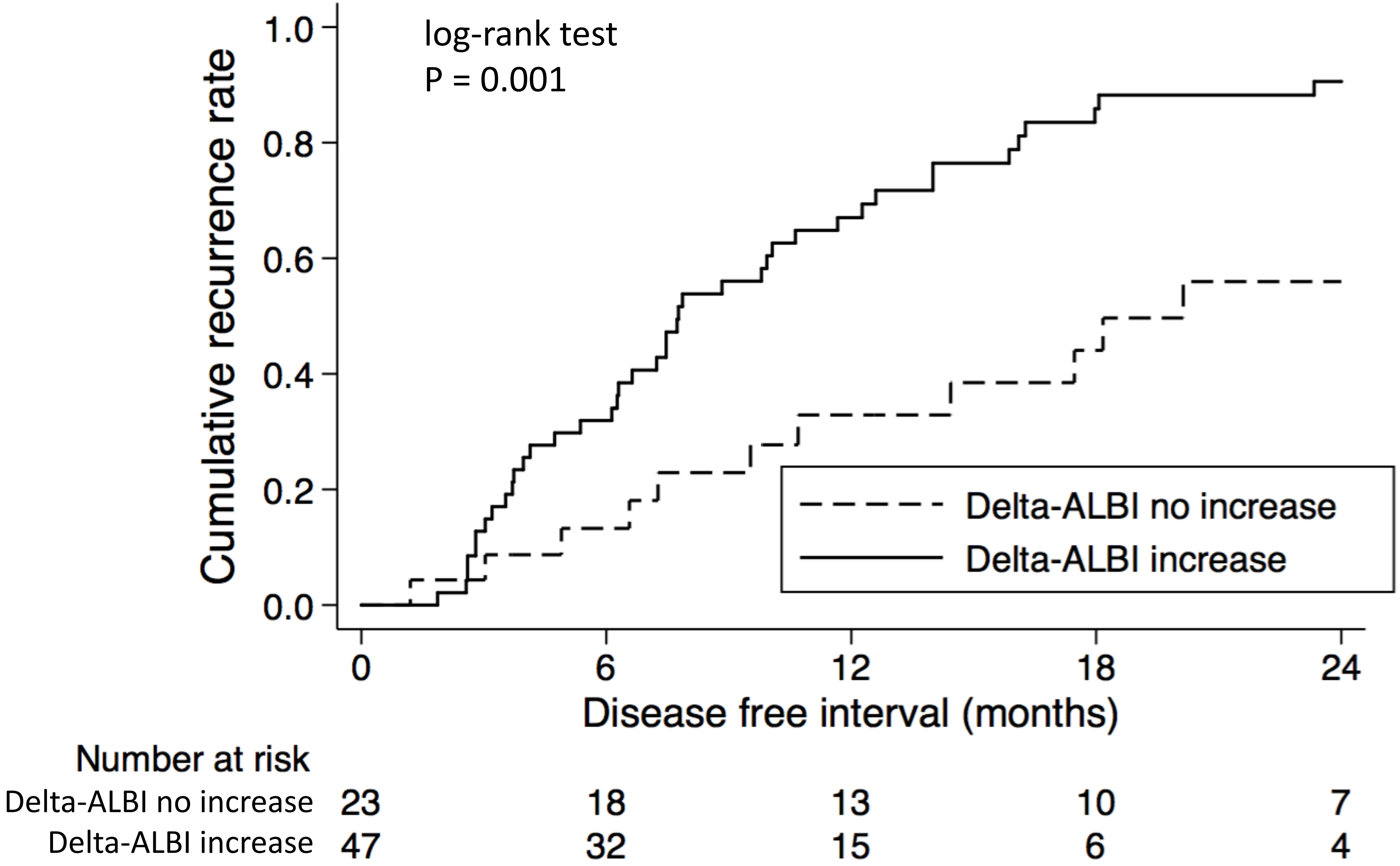

Supplementary Figure S2B.

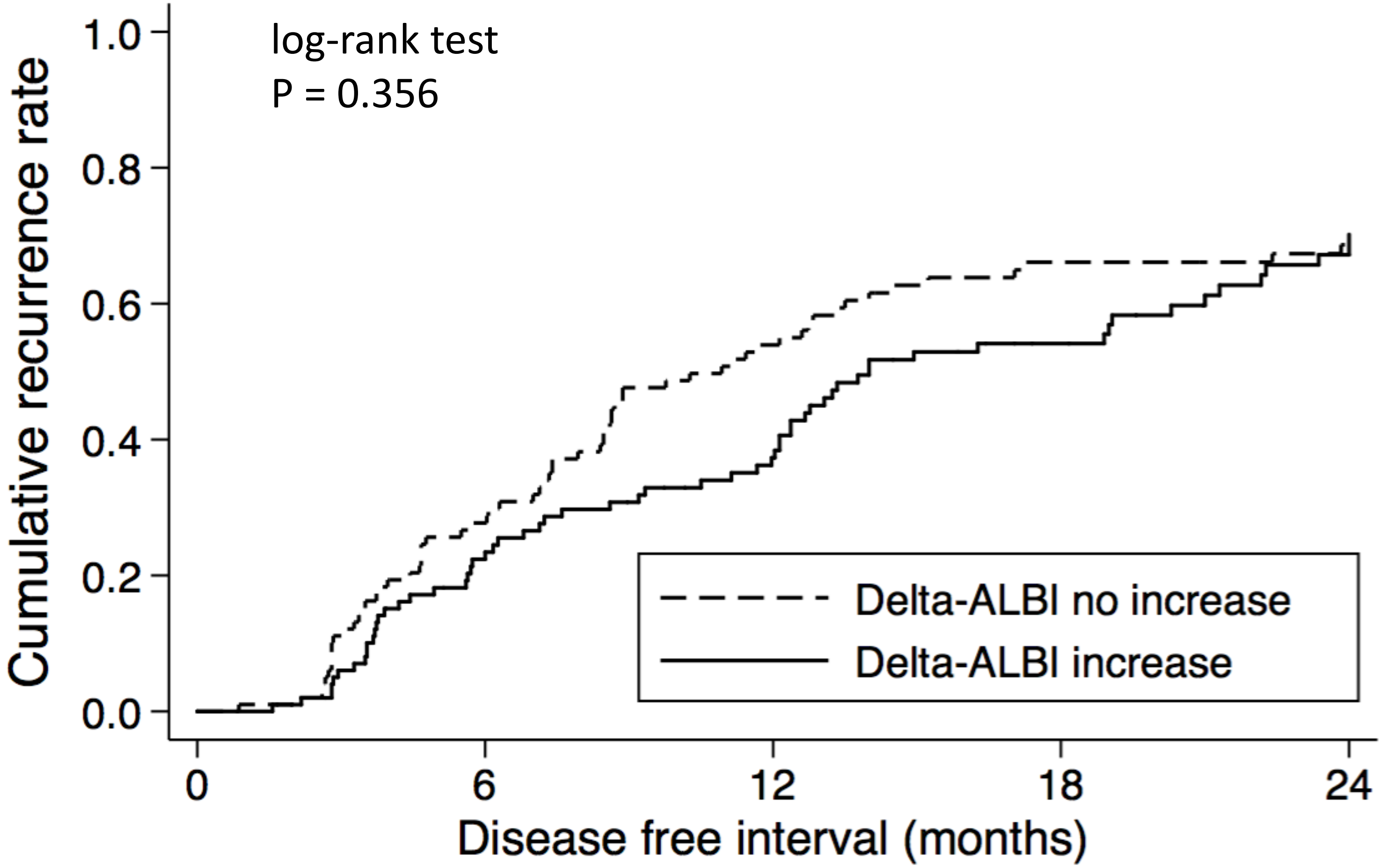

Number at risk	0	6	12	18	24
Delta-ALBI no increase	99	69	43	30	24
Delta-ALBI increase	102	74	57	34	22
